# Therapeutics

**Published:** 1876-01

**Authors:** 


### VI. Therapeutics.

1. *Salicine in Diarrhœa.* Vail. (*N. Y. Med. Jour.*, Oct., 1875.)

After detailing a few cases of severe diarrhœa treated with marked success with the above named drug, the writer remarks :

"It combines the tonic element of quinine with strong astringent properties. In cases of enteric atony, especially in chronic cases, from chronic diarrhœa or other debilitating diseases, where quinine is inadmissible from its tendency to irritate the bowels, in my hands salicine has given the most flattering results. My experience has been small, and it is the design of this article to draw attention to this subject, that we may have further light from more experienced quarters. So far, our authorities, as the Dispensatory, Stillé, Flint, and West, make, if any, the most cursory note of salicine as a medicine. Only in Reynolds's 'System of Medicine' does Dr. Aitken mention it as 'a remedy which has been found very valuable in cases of chronic diarrhœa, where opiates and astringents have entirely failed.' I always prescribed it in powders, to be given in sugar and water. You will be surprised how readily it will be taken by the little ones ; much more readily than quinine, though more bitter. It seems to be a purer bitter, and one much more acceptable to the stomach. In no case was it ejected till the system seemed satisfied. It is my rule to continue its administration for from two weeks to two months after the subsidence of the disease proper, according to the time the disease has existed. The dose corresponds with that of quinine."

2. *Anti-Neuralgic Properties of Essence of Mint.* Délioux de Savignac. (*Am. Jour. Dent. Sci.*, Nov., 1875.)

The essence of mint exerts a special influence upon the sensory nerves, which diminishes the abnormal vivacity of their reactions when these become exalted in painful affections. It is comparable, in this respect, to chloroform, ether and camphor. Though its high price will prevent its being extensively used in the pure state as an anti-neuralgic and anti-rheumatic, it may be employed in alcoholic solution as an application in the form of liniment; and camphor, chloroform and laudanum may be added to it with advantage in various cases. Dr. Savignac has found it extremely useful in relieving the pain of pruritus ; in alleviating, when employed in the form of injections, the miseries of vulvo-vaginal hyperæsthesias, vaginismus, and neuralgias of the head and face, which are best treated by making a small ball of wool of the size of a nut, which has been made to imbibe a few drops of the essence, and rubbing the painful part with it gently. It is then to be covered with a larger piece of wool, and the whole kept in position for a few minutes with the palm of the hand. The success of these external applications of the essence of mint in neuralgias of the head and face, and even in cases of congestive cephalalgia, has been both prompt and permanent. He adds, however, that the cause of the pain must be local for the cure to be permanent, since, if it be dependent on hysteria, or be connected with intermittent fever, or with gastric derangement, the primary cause must first be removed. The remedy seems to have been long known in China. For intermuscular and parenchymatous neuralgias, which are especially obstinate, he has tried the following as a hypodermic injection: Hydrochlorate of morphia, 1¼ grains ; mint water, 140 minims; alcoholate of mint, 15 minims. Mix.

3. *Administration of Medicine to Infants Through the Mother's Milk.* Lewald. (*Lyon Médicale*, Oct., 1875.)

The elimination, by the milk of the mother, of iron, bismuth, iodine, and its compounds, arsenic, lead, zinc, antimony, mercury, alcohol, and several narcotics, is here considered. Numerous experiments were made in the goat. The principal conclusions arrived at are : 1. A larger quantity of iron can be administered to the infant through the mother's milk than by any other means. 2. Bismuth likewise is eliminated by the milk, but in very small quantity. 3. Iodine does not appear in the milk until ninety-six hours after taking it; the iodide of potassium, given in doses of forty grains *per diem*, appears four hours after ingestion, and continues to be eliminated for eleven days. 4. Arsenic appears in the milk at the end of seventeen hours, and its elimination had not ceased after sixty hours. 5. Though one of the most insoluble preparations, the oxide of zinc is nevertheless eliminated by the milk, and it is probable that this is also the case with the other preparations of zinc; fifteen grains of oxide of zinc were found in the milk at the end of from four to eight hours, and it disappears sooner than iron, because no trace of it could be discovered after fifteen or sixteen hours. 6. The elimination of antimony is an undeniable fact, and it is well to bear this in mind during the period of nursing. The same holds true in regard to mercurial preparations. 7. That alcohol and the narcotics are eliminated by the milk has not been demonstrated. 8. Sulphate of quinine is eliminated very easily; a child suffering from intermittent fever was cured by administering quinine to the nurse.

4. *Treatment of Chronic Tumefaction of the Spleen.* MOSLER, of Greifswald. (*Deuts. Arch. f. kl. Med.; N. Y. Med. Jour.*, Oct., 1875.)

Provided certain precautions are taken, injections into the spleen can be made without danger. It is first necessary to diminish the quantity of blood in the organ, and this object is attained by giving an hypodermic injection of hydrochlorate of quinine. For some time before the parenchymatous injection is made, ice is applied over the spleen, and as soon as the organ has contracted so that its inferior extremity lies against the abdominal wall, the injection can be made ; if it causes much pain, it can be followed by an injection of morphine. Carbolic acid (1-200) was first employed; in another case, Fowler's solution; and these injections did no harm. In one of the cases, the diminution of the volume of the organ was considerable. One cubic centimetre of a mixture of Fowler's solution (1-10) was injected several times. The pain was relatively moderate, the cold being kept applied over the splenic region. This treatment caused complete cure in a patient after all other means had failed.

